# Differential associations of febuxostat and allopurinol with incident and worsening frailty risk in nephrology patients

**DOI:** 10.7150/ijms.128982

**Published:** 2026-08-04

**Authors:** Szu-Ying Lee, Jui Wang, Kyung Don Yoo, Chia-Ter Chao, Kuo-Liong Chien, Kuan-Yu Hung

**Affiliations:** 1 Division of Nephrology, Department of Internal Medicine, National Taiwan University Hospital Yunlin Branch, Yunlin County, Taiwan.; 2 Institute of Epidemiology and Preventive Medicine, College of Public Health, National Taiwan University, Taipei, Taiwan.; 3 Health Data Research Center, National Taiwan University, Taipei, Taiwan.; 4 Division of Nephrology, Department of Internal Medicine, Ulsan University Hospital, University of Ulsan College of Medicine, Republic of Korea.; 5 Basic-Clinical Convergence Research Institute, University of Ulsan, Republic of Korea.; 6 Division of Nephrology, Department of Internal Medicine, National Taiwan University Hospital and National Taiwan University College of Medicine, Taipei, Taiwan.; 7 Graduate Institute of Toxicology, National Taiwan University College of Medicine, Taipei, Taiwan.; 8 Graduate Institute of Medical Education and Bioethics, National Taiwan University College of Medicine, Taipei, Taiwan.; 9 Division of Nephrology, Department of Internal Medicine, Min-Sheng General Hospital, Taoyuan City, Taiwan.

**Keywords:** allopurinol, chronic kidney disease, frailty, gout, hyperuricemia, uric acid

## Abstract

**Background:**

Hyperuricemia and gout are linked to adverse outcomes and may influence frailty risk, but evidence on the impact of uric acid (UA)-lowering therapy on frailty among nephrology patients is limited. We evaluated the association of febuxostat and allopurinol use with incident and worsening frailty in a large nephrology patient cohort.

**Methods:**

This retrospective cohort study included 19,389 adults attending nephrology clinics at a tertiary medical center in Taiwan between 2013 and 2021 with available UA and estimated glomerular filtration rate (eGFR) data. Exposure was the use of febuxostat, allopurinol, or both agents versus no UA-lowering therapy. We examined incident frailty and worsening frailty defined by the FRAIL scale after follow-up. Kaplan-Meier and Cox proportional hazards models were applied, adjusting for demographics, comorbidities, medications, baseline frailty, and laboratory data.

**Results:**

Over a median follow-up of 3.0 years, 892 (4.6%) participants developed incident frailty and 5,253 (27.1%) had worsening frailty. After adjustment, febuxostat use was associated with higher probability of incident frailty (hazard ratio (HR) 1.22, 95% confidence interval (CI) 1.01-1.48), while allopurinol use was associated with lower probability (HR 0.65, 95% CI 0.46-0.93) compared to non-users. Both users had no alterations in incident frailty probability. Neither therapy was significantly associated with worsening frailty. Subgroup analyses showed modification effects from age and eGFR levels.

**Conclusions:**

Among nephrology patients, UA-lowering therapy showed divergent associations with frailty probability: febuxostat use correlated with higher whereas allopurinol use correlated with lower incident frailty probability, with no effect on frailty progression. Choice of UA-lowering therapy may have implications for functional outcomes in this high-risk population.

## Introduction

The burden of hyperuricemia and its complications have become a major public health concern. From an evolutionary perspective, uric acid (UA) exerts dual effects: while it sustains blood pressure regulation and possesses anti-oxidant properties, excessive UA precipitates and crystallizes, triggering local and systemic inflammation 1. Data from various countries indicate that the prevalence of hyperuricemia in the general population ranges from 16% to 18.8%, with higher rates among men, older individuals, and those living in certain geographic regions 2, 3. Global Burden of Disease 2021 estimates that gout, an important complication of hyperuricemia, affects 55.8 million people worldwide, with a projected 72% increase by 2050 4. Gout also accounts for 20.5 years lived with disability per 100,000 population globally, partly due to co-existing high body mass index (BMI) and chronic kidney disease (CKD).

Existing evidence links hyperuricemia and gout to a broad range of adverse outcomes. Asymptomatic hyperuricemia may increase the risk of cardiovascular diseases, including hypertension, atherosclerosis, heart failure, kidney function decline, and nephrolithiasis 5, 6. Meta-analyses show that gout increases mortality from cardiovascular, infectious, and gastrointestinal causes by 24% to 42%, as well as overall mortality by 23% 7. These associations may reflect the deleterious effects of UA itself demonstrated in experimental work, mechanisms that promote UA elevation and decreased solubility, and coexisting morbidities such as hypertension, diabetes, and CKD 8. These observations have prompted investigation into UA-lowering therapies to improve outcomes. However, therapeutic efficacy appears to vary by patient characteristics, comorbidities, the types of UA-lowering agent, and achieved UA targets, and the outcomes examined. Although some studies report modest reductions in cardiovascular risk with pharmacologic UA reduction 9, others show neutral effects 10. Similar findings in kidney outcomes are also reported by network meta-analysis 11.

Existing literature suggests that higher UA is associated with biological aging and the development of degenerative phenotypes. A biobank study disclosed that hyperuricemia contributed to epigenetic age acceleration in addition to lifestyle factors 12. Population-based cohorts from the East and the West indicate that for every 1 mg/dL UA increase, there is a 0.15 to 0.38 increase in allostatic load, physiologic wearing in response to stress 13. In addition, hyperuricemia and gout may also affect musculoskeletal health. Elevated UA have been implicated in muscle, joint, and bone degeneration through inducing oxidative stress, crystal deposition, and inflammation 14. Preliminary studies suggest that UA-lowering therapies, particularly allopurinol, may reduce oxidative stress and local inflammation in muscle, potentially facilitating recovery after injury 15. Because musculoskeletal dysfunction affects functional status, UA levels and UA-lowering therapies may potentially influence physical performance and other degenerative syndromes associated with chronological aging, such as frailty.

Frailty, a geriatric syndrome describing an individual's increased vulnerability and the associated adverse outcome influences, exhibits a high prevalence in older adults and patients with CKD. A meta-analysis of 15 studies identified that 37.9% and 38.1% patients with CKD have pre-frailty and frailty, respectively, rising further among those with end-stage kidney disease 16. Frailty itself also increases the risk of acute kidney injury by 2-fold 17, exemplifying its importance in kidney patients. The development of frailty in this population is associated with cardiovascular, metabolic, musculoskeletal, and psychiatric complications, leading to impaired quality of life and worse functional performance 18. Pathogenesis of and contributors to frailty in patients with CKD include uremic toxins, metabolic derangements, insulin resistance, and effects from associated comorbidities 19, 20. Among these factors, UA can be an under-recognized one. Indeed, higher serum UA has been shown to be an independent risk factor for frailty among older adults 21. Higher UA levels are also shown to be independently correlate with frailty among patients with an estimated glomerular filtration rate (eGFR) <60 mL/min/1.73m^2^, especially younger ones 22. However, very few studies have examined the relationship between UA-lowering therapies and frailty risk in patients with hyperuricemia or gout, despite the perceived importance of UA in the pathogenesis of frailty in patients with CKD. Emerging studies support the potential benefits of UA-lowering treatments against kidney function deterioration in those with CKD and hyperuricemia 23, but whether such treatments ameliorate geriatric syndromes like frailty in this population remains unclear. We therefore hypothesized that UA-lowering therapies might affect frailty risk. Given that CKD and other kidney disorders increase the likelihood of abnormal UA levels, we used a large cohort of nephrology patients to test this hypothesis.

## Methods

### Ethical statement

The protocol of this study belonged to the parent study investigating the epidemiology of cystic kidney disease and nephrology patients, approved by the institutional review board of National Taiwan University Hospital (NTUH; NO. 202205024RINA). Informed consent was waived because all data were de-identified prior to analysis. The study complied with the Declaration of Helsinki.

### Cohort assembly

**Figure 1:**
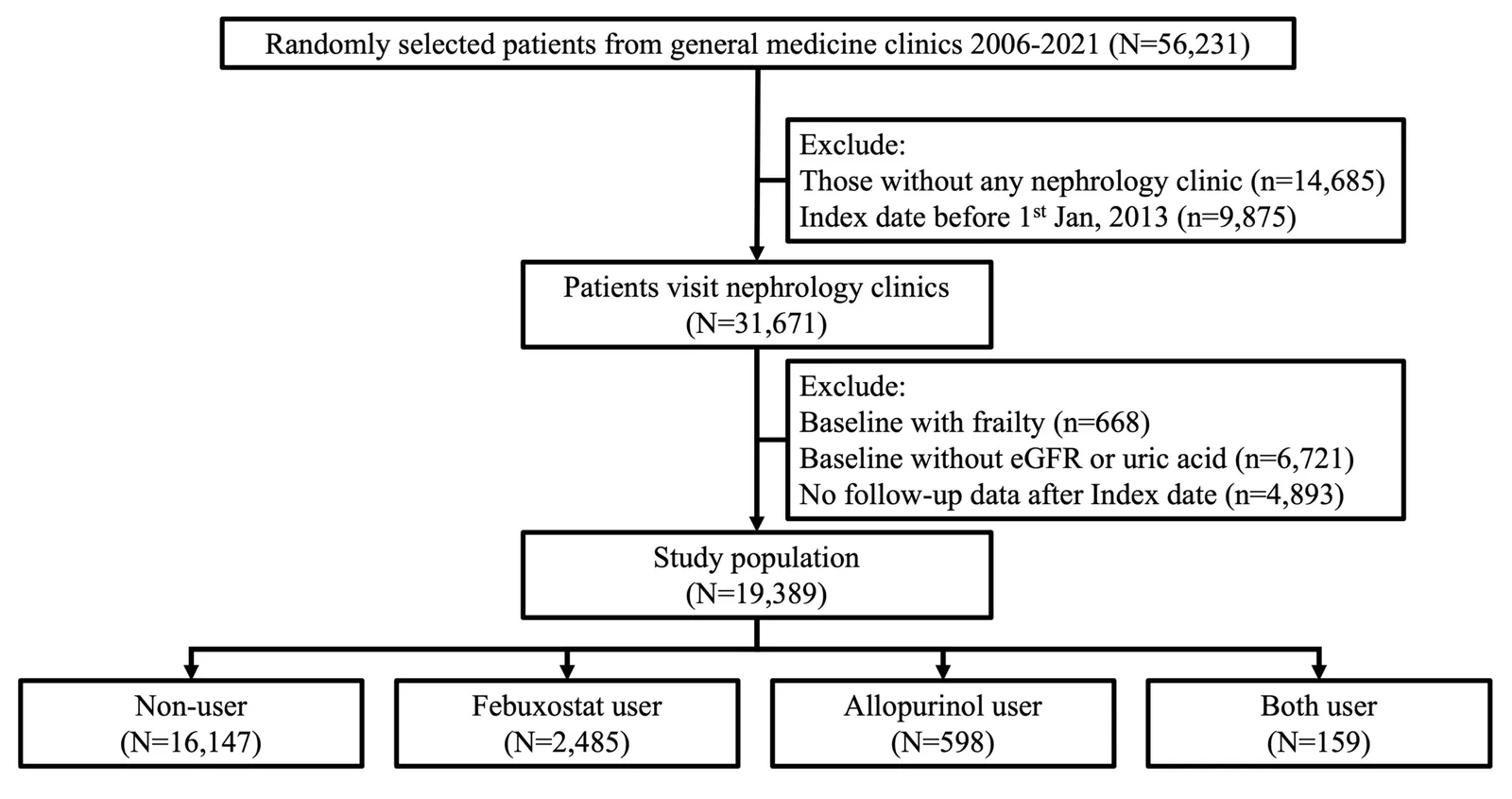
The algorithm for identifying study participants*. eGFR, estimated glomerular filtration rate*.

We identified patients from the NTUH integrated Medical Database (NTUH-iMD) who attended general medicine clinics between 2006 and 2021. We excluded individuals who (i) never attended a nephrology clinic, (ii) first visited a nephrology clinic before January 1, 2013, (iii) had frailty, the primary outcome of interest, before the index date, (iv) lacked available eGFR or UA data, or (v) had no follow-up after the index date (**Fig. 1**). Since febuxostat received its licensing for reimbursement in Taiwan since late 2012, we chose January 1, 2013 for aligning all UA-lowering treatments timings. The index date was defined at one year after the first nephrology clinic visit. Patients were followed up from the index date, until the occurrence of primary outcomes (explained below), death, or the last recorded visit at NTUH, whichever occurred first.

### Data collection

We collected demographic data (age, sex), BMI, comorbidities (hypertension, diabetes, hyperlipidemia, CKD, cardiovascular disease, hyperuricemia, and kidney stones), and medications (blood pressure-lowering, anti-diabetic, lipid-lowering, antiplatelet, anticoagulants, UA-lowering, and non-steroidal anti-inflammatory drugs (NSAIDs). Blood pressure-lowering drugs were further divided into six types (angiotensin-converting enzyme inhibitors, angiotensin receptor blockers, calcium channel blockers, diuretics, β- and α-blockers), since some of these medications might affect hyperuricemia incidence. Medication use was defined as receipt of medication for more than one month within the year before the index date. Laboratory data included UA and serum creatinine; eGFR was calculated using the Modification of Diet in Renal Disease (MDRD) formula.

### Exposure groups

We divided participants according to their UA-lowering regimens, into those who did not receive any such medications (non-users), febuxostat users, allopurinol users, or concurrent users before the index date. Exposure to febuxostat or allopurinol was defined as having received prescriptions for more than 1 month within the year before the index date. To further characterize medication utilization, we calculated the cumulative prescription days of each UA-lowering agent during the one-year period before index date and included these data in the dose-response analyses. We did not include benzbromarone users in this study, since the proportion of these patients in nephrology clinics is low, as reported by others 24.

### Outcomes

The primary outcomes were incident frailty and worsening frailty during follow up. Frailty was defined by the fatigue, resistance, ambulation, illness, and loss of weight (FRAIL) scale, a well-established frailty-assessing instrument 25 among older adults and individuals with chronic diseases. Our approach for operationalizing FRAIL scale has been published previously 26. FRAIL scale contains five dimensions, with one point assigned to each positive dimension, which was assessed based on vital diagnostic groupings and procedures during ≥1 hospitalization or ≥2 out-patient clinic episodes during the preceding years of the index date. The fatigue component was identified based on international classification of disease (ICD) diagnoses with close proximities to keywords including malaise, fatigue, asthenia, and general weakness. For resistance component, difficulty in stair climbing was identified using physical deconditioning, falls and associated complications treatments, and debility related coding. For ambulation component, we identified gait abnormalities and conditions with walking difficulty for operationalization. For loss of weight component, we used diagnoses or procedures for malnutrition, its complications and management for identification. The illness component was based on the original scheme. A complete list of diagnosis or procedures extracted can be found elsewhere 26. Individuals with ≥3 out of a total 5 points are deemed frail, whereas those with ≥1-point increase during follow-up compared to baseline were considered to have worsening frailty. A recent systematic review concludes that an increase of 1.8 FRAIL scores is equipercentile to 0.25 increase in the frailty index score 27. We previously also demonstrated that FRAIL-identified frailty significantly increased mortality and aggravated cardiovascular outcomes among those with diabetes and CKD, with incremental adverse influences paralleling rising FRAIL scores 26, 28. Correlations between our FRAIL scale and frailty index scores are demonstrably good 29.

### Statistical analysis

Continuous variables are summarized as means ± standard deviations and categorical variables as counts with percentages. Group comparisons for all variables (demographic, physical, comorbidities, baseline frailty assessment results, medications, and laboratory data) used one-way analysis of variance (ANOVA). After follow-up, we recorded these patients' cumulative incidence of primary outcomes and incident densities. Kaplan-Meier event-free analyses were utilized to evaluate incident and worsening frailty event in each group, compared using log-rank tests. Cox proportional hazards models were then performed to analyze the relationship between UA-lowering therapies and outcome risk, adjusting for demographic, physical data, comorbidities, medications, baseline FRAIL results, and eGFR/UA levels. Hazard ratios (HRs) with 95% confidence interval (CI) were reported. Proportional hazards assumptions were evaluated using Schoenfeld residuals. We further performed subgroup analyses based on age, sex, and eGFR levels. In addition, another set of analysis using inverse probability of treatment weighting (IPTW) was done to reduce confounding influences, with propensity scores estimated using demographic characteristics, physical profiles, comorbidities, medication use, and laboratory data. A negative control outcome (hospitalization for urinary tract infection) analysis was also done to reassure our result validity. Dose-response analyses were further done based on the duration of the medication use (febuxostat or allopurinol) in comparison with the non-user group. We used the median duration of the index medication used as the cutoff for grouping purpose. Finally, several sensitivity analyses were performed. First, we adjusted for the types of blood pressure-lowering drugs, instead of blood pressure-lowering drug use or not, in order to account for their individual influences. Second, we replaced eGFR data calculated from MDRD formula with those from the chronic kidney disease epidemiology collaboration (CKD-EPI) formula during regression analyses. At last, we performed Fine-Gray modeling using mortality as a competing risk event. In all analyses, a two-sided *p* < 0.05 was considered statistically significant.

## Results

Totally 56,231 patients were randomly selected from general medicine clinics of our institute during the study period (2006-2021) (**Fig. 1**). After applying the exclusion criteria, we identified 19,389 nephrology patients, dividing them into non-users (83.3%), febuxostat (12.8%) or allopurinol (3.1%) users, and both users (0.8%) (**Fig. 1**).

The mean age of enrolled participants was 64.7 ± 15.6 years, with 53.4% older adults (≥ 65 years) (**Table 1**). Hypertension was present in 81.5% participants, but only one-third (33.1%) had cardiovascular diseases. About 42% participants had hyperuricemia. Their mean eGFR and UA levels were 57.83 ± 37.57 mL/min/1.73m^2^ and 6.4 ± 2.25 mg/dL, respectively (**Table 1**). They were evenly distributed regarding the proportion of CKD stage 1 to 5. Compared with non-users, UA-lowering therapy users (febuxostat, allopurinol, and both groups) were significantly older, more often male, and higher comorbidity burden and medication use (**Table 1**). UA-lowering therapy users had significantly higher FRAIL scale scores and lower eGFR levels than non-users. Febuxostat users had the lowest UA levels than the other three groups (**Table 1**).

**Figure 2:**
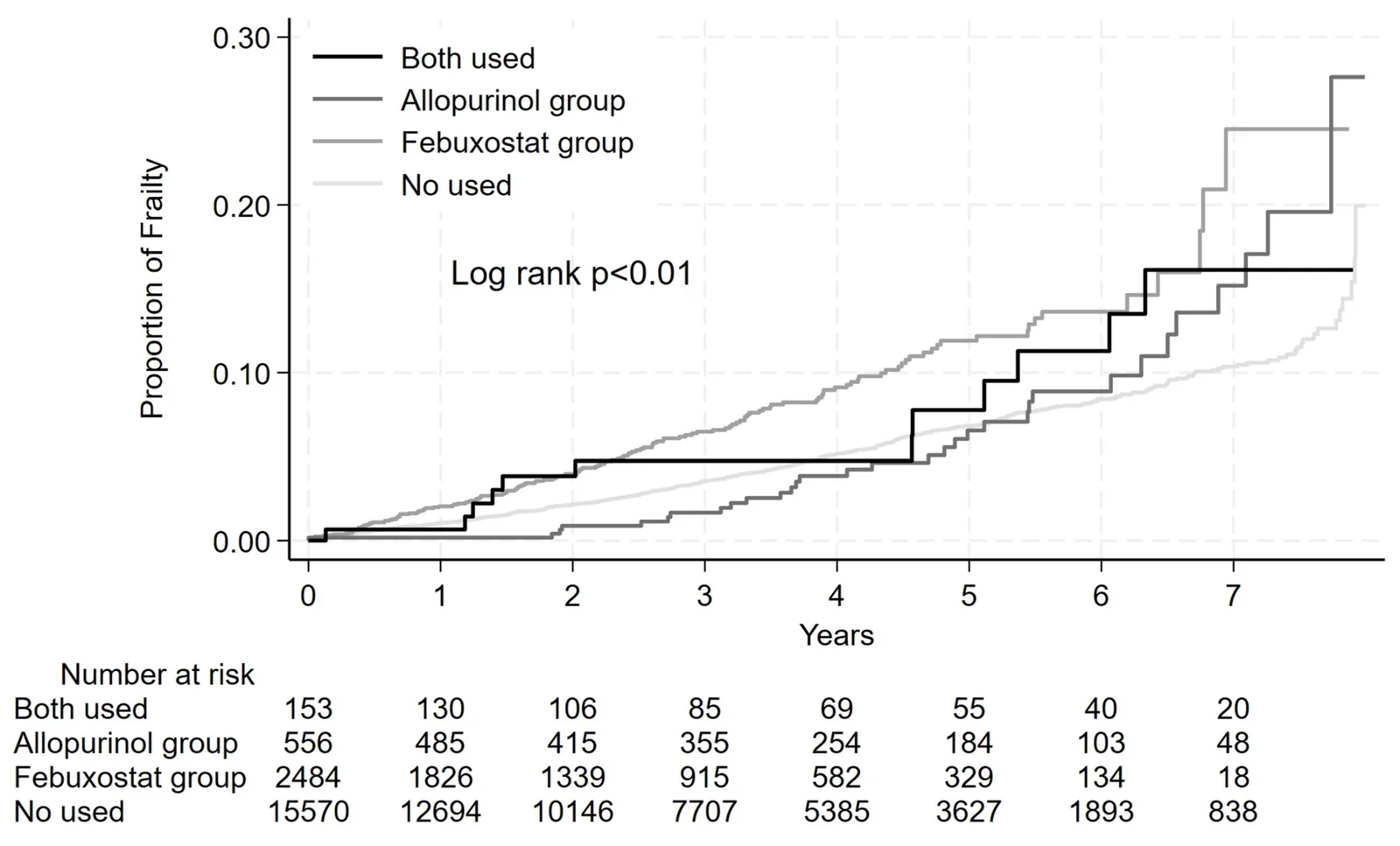
Incident frailty-free event curves of different uric acid-lowering therapy groups.

**Figure 3:**
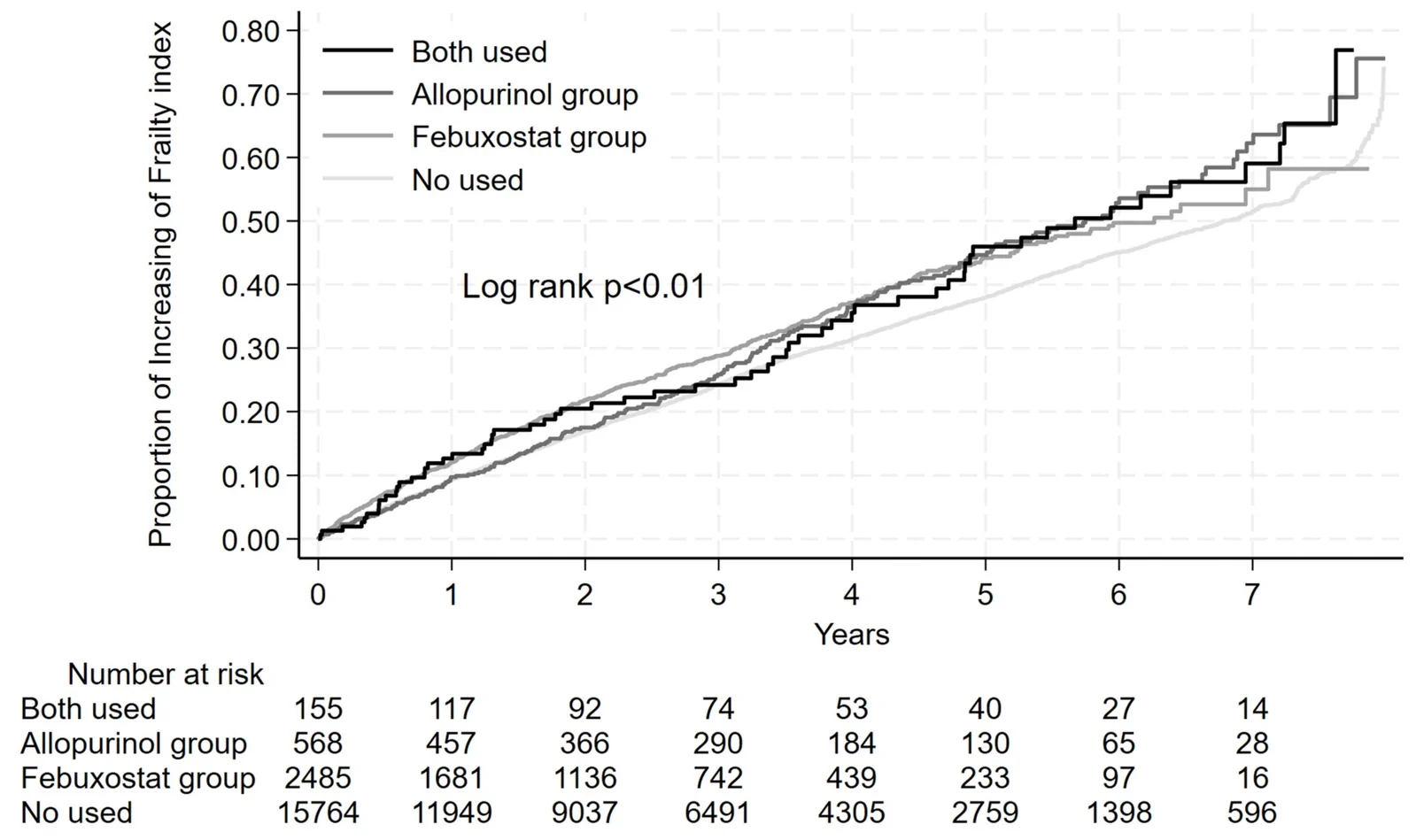
Worsening frailty-free event curves of different uric acid-lowering therapy groups.

During a median follow-up of 3 years (interquartile range, 1.38 - 4.98), 892 participants (4.6%) developed incident frailty and 5,253 (27.1%) experienced worsening frailty (**Tables 2** and **3**). Kaplan-Meier analyses showed the lowest incidence of frailty among non-users, whereas febuxostat users had higher frailty incidence (**Fig. 2**). Non-users also had the lowest incidence of worsening frailty (**Fig. 3**). The incidence density of frailty development (**Table 2**) and worsening frailty (**Table 3**) was highest among febuxostat users compared to the other three groups. In unadjusted Cox models, febuxostat users had a higher probability of developing incident frailty (HR 1.992, 95% CI 1.668-2.381) compared with non-users, whereas allopurinol and both users did not (**Table 2**).

After adjustment, febuxostat use remained significantly associated with higher probability of developing incident frailty (HR 1.221, 95% CI 1.010-1.477), while allopurinol use was associated with a lower probability (HR 0.652, 95% CI 0.455-0.933) (**Table 2**). No significant difference in probability was observed in the both user group. In this analysis, the proportional hazards assumption was examined using Schoenfeld residuals, and the global test returned a p-value of 0.7313, suggesting that the assumption was not violated. Cox proportional hazard regression with IPTW revealed that febuxostat users had significantly higher probability of incident frailty (HR 1.604, 95% CI 1.462-1.760) (**Supplementary Table S1**). Negative control outcome analyses focusing on urinary tract infection showed that neither febuxostat nor allopurinol affect the probability of such event (**Supplementary Table S2**).

As for worsening frailty, unadjusted analysis showed a significantly higher probability among febuxostat users compared to non-users (HR 1.307, 95% CI 1.203-1.420) (**Table 3**). However, the associations no longer existed after multivariate adjustment (HR 1.051, 95% CI 0.962-1.148). Allopurinol alone or both users did not have differences in probabilities from non-users. In this analysis, the proportional hazards assumption was examined using Schoenfeld residuals, and the global test returned a p-value of 0.4497, suggesting that the assumption was not violated. Cox proportional hazard regression with IPTW revealed that febuxostat users had significantly higher probability of worsening frailty (HR 1.045, 95% CI 1.003-1.089) (**Supplementary Table S1**).

Exploratory subgroup analyses showed that the associations between UA-lowering treatment and the probability of developing incident frailty were significant predominantly among participants ≥ 65 years and those with an eGFR < 45 or < 30 mL/min/1.73m^2^ (**Table 4**). Febuxostat users ≥ 65 years had significantly higher probability of developing incident frailty than non-users of the same age stratum, whereas allopurinol users had lower probability. Among those with low eGFR levels, only allopurinol users had a lower probability of developing incident frailty than non-users (**Table 4**). However, several strata contained limited numbers of outcome events, and these findings should therefore be interpreted cautiously.

We next examined the influence of cumulative drug duration use on the probability of outcome development. In exploratory analyses stratified according to cumulative pre-index prescription duration, Febuxostat users for longer than the median duration (e.g. 161 days) demonstrated a higher probability of incident frailty (HR 1.287, 95% CI 1.008-1.643) than non-users, followed by febuxostat user of shorter duration (p for trend, 0.0287) (**Supplementary Table S3**). Allopurinol users for longer than the median duration (e.g. 140 days) had significantly lower probability of incident frailty (HR 0.626, 95% CI 0.395-0.992) than non-users, followed by allopurinol user of shorter duration (p for trend, 0.0351) (**Supplementary Table S3**). However, these findings should be interpreted cautiously because duration-based categorization may be susceptible to immortal time bias and does not account for treatment modifications during follow-up. Sensitivity analyses were also performed to account for the influences of blood pressure-lowering drug types, eGFR derivation formula, and the competing influence of mortality in our findings. After adjusting for blood pressure-lowering drug types, allopurinol users still had significantly lower probability of incident frailty (HR 0.642, 95% CI 0.448-0.919), whereas febuxostat users had marginally higher probability (HR 1.183, 95% CI 0.977-1.433) (Model 2; **Supplementary Table S4**). Both users similarly had no difference in incident frailty probability compared to non-users. If we replaced the MDRD formula with CKD-EPI for eGFR calculation, the results showed that allopurinol users had significantly lower probability of incident frailty (HR 0.638, 95% CI 0.445-0.913), whereas febuxostat users had marginally higher probability (Model 3; **Supplementary Table S4**). Finally, Fine-Gray modeling with mortality as a competing event yielded similar findings (Model 4; **Supplementary Table S4**).

## Discussion

In this large cohort of nephrology patients, we investigated whether the types of UA-lowering therapies affected their probability of incident and worsening frailty. Febuxostat use was independently associated with an increased probability of developing incident frailty after 3 years, whereas allopurinol use was associated with a reduced probability, after extensive confounder adjustment. Neither agent use was associated with frailty progression over time. These findings can potentially guide our selection of UA-lowering regimens when confronting patients with therapeutic indication. UA-lowering therapy choice may affect functional outcomes beyond urate control, particularly in the aging population or those at high frailty risk, since frailty constitute an important consideration in geriatric care.

Although we did not specifically define hyperuricemia in this study, UA-lowering therapies are usually prescribed to manage hyperuricemia and/or gout from a clinical perspective. If we used the proportion of receiving therapy as a surrogate for hyperuricemia presence in our cohort, the disease prevalence (16.7%) is compatible with data from the existing studies 2, 3 and local report 30. Other researchers discovered that patients with hyperuricemia had higher BMI, blood pressure, and poorer kidney function compared to those without, whereas fasting glucose and CKD prevalence were higher in older ones 31. Our UA-lowering therapy users thus exhibited similar physical, comorbidity patterns, and laboratory features relative to non-users (**Table 1**), supporting the potential of extrapolating our findings to other populations with hyperuricemia.

The independent associations between specific UA-lowering agent use and incident frailty probability is interesting. Several reasons may be responsible for this phenomenon. In the Cardiovascular Safety of Febuxostat and Allopurinol in Patients with Gout and Cardiovascular Morbidities (CARES) trial, febuxostat users had a modest albeit significantly higher risk of cardiovascular mortality than allopurinol users 32. Since cardiovascular complications are key contributors to incident frailty 33, such adverse influences may play a role in the relationship between febuxostat use and frailty probability. Evidence also suggests that allopurinol use lowers the cardiovascular risk in patients with hyperuricemia 34, partly contributing to the observed allopurinol-associated benefits in frailty probability attenuation. In addition, pharmacokinetic studies imply that febuxostat potently increases the effective concentrations of multiple drugs through inhibiting their metabolizing enzymes, whereas allopurinol does not 35. This phenomenon can lead to unexpected adverse effects emerging from febuxostat-containing polypharmacy, an established frailty risk factor 36. This is particularly of concern, since patients with hyperuricemia or gout tend to have multiple morbidities. Finally, a reno-protective effect associated with allopurinol has been reported, compared to febuxostat among patients with hyperuricemia 37. This can also be translated to a reduction in frailty probability we observed.

Importantly, our study included a non-user comparator group, demonstrating that the potential benefits associated with allopurinol and adverse influences associated with febuxostat on incident frailty are additive rather than relative to one another. The absence of associations with worsening frailty suggests that higher UA-lowering therapy strength may influence frailty onset rather than progression in this population. However, confounding by indication remains an important limitation. Patients prescribed febuxostat or allopurinol differed from non-users in multiple clinical aspects, and these factors may have influenced both treatment selection and frailty risk. Therefore, our observations should not be interpreted as firm evidence of causal treatment effects.

Interpretation of our subgroup analyses warrants caution. Several subgroup strata contained relatively small numbers of outcome events, with some categories having fewer than 10 events and others having no observed events. Under these circumstances, hazard ratio estimates may be unstable and associated with substantial statistical uncertainty. Accordingly, these analyses should be regarded as exploratory and hypothesis-generating rather than confirmatory evidence of effect modification. Larger studies with adequate event numbers will be necessary to validate potential subgroup-specific associations.

Our findings have several clinical implications. Prescribing UA-lowering therapy in nephrology care typically balances multiple indications, including gout control, urolithiasis, and potential cardiovascular or kidney benefits. The use of xanthine oxidase inhibitors, including allopurinol and febuxostat, particularly suits the purpose of attenuating oxidative stress in cardiac tissues and vasculature, through which organ protection effects are expected to generate 38. However, based on our results, clinicians must further refine their therapeutic drug of choice by broadening the considerations to include effects on incident frailty, a looming threat within geriatric care. Our findings therefore tip the balance toward the use of allopurinol for managing hyperuricemia and/or gout, in patients at a higher frailty risk.

There are strengths and limitations for our study. Ours is among the first few to investigate the influences of UA-lowering therapies on the probability of frailty. We assembled a moderately large cohort of nephrology patients to answer this question, and adjusted for multiple covariates to obtain results. The outcomes of interest, incident and worsening frailty over time, have been tested repetitively by others 29, 39, with validity reassured by cross-referencing with frailty index results. Nonetheless, several limitations warrant consideration. First, this is a retrospective cohort study, limiting its ability to derive unbiased findings. To minimize these potential biases, we applied IPTW, performed multivariate adjustments using different statistical models, and conducted competing risk analyses to account for death as a competing event. Second, our participants came from nephrology clinics, a feature that renders result applicability to those with other morbidities (such as cardiac or pulmonary ones) unclear. Third, our patients are mostly Han Chinese in origin, which may limit generalizability. Genetic factors such as HLA-B*5801 prevalence may influence therapy choice (allopurinol vs. febuxostat) and potentially the outcomes of interest. Anecdotal studies suggest that genetic polymorphisms can play a role in affecting frailty susceptibility in middle age to older adults 40. The incidence and risk factors contributing to frailty may vary substantially across healthcare settings and care scenarios 41, further posing influences on our results. An additional limitation relates to the exploratory duration-response analyses. Categorization according to cumulative treatment duration before the index date may introduce immortal time bias. Consequently, this part of analyses should be considered hypothesis-generating, and future studies employing time-dependent analytic approaches are warranted. Finally, unmeasured confounders such as dietary patterns, physical activity, and social factors, which might affect frailty risk were unavailable in our database. Imbalances in physical activity levels, for example, may correlate with frequent gouty experiences associated with hyperuricemia or osteoporosis, affecting frailty incidence in at-risk populations 42. Dietary preferences for foods with high glycemic index can lead to dysglycemia and complications associated with diabetes, all of which can modulate the risk of frailty 43. Prospective studies are needed to confirm these associations and clarify the mechanisms underlying our findings.

## Conclusion

In this large, nephrology patient-based cohort, we found that the choice of UA-lowering therapy was differentially associated with the probability of incident frailty. Febuxostat use was independently associated with a higher probability of incident frailty, whereas allopurinol use was associated with a lower probability; neither agent was consistently associated with frailty progression. These observational findings should be interpreted cautiously given the potential for confounding by indication and require prospective validation before being used to guide treatment selection. Nonetheless, the possibility exists that the choice of UA-lowering therapy may bear functional relevance beyond urate control, particularly in patients at high risk of frailty.

## Supplementary Material

Supplementary tables.

## Figures and Tables

**Table 1:** Baseline characteristics of enrolled patients

	Total population (n = 19,389)	Non-users (n=16,147)	Uric-acid lowering treatment groups			P value*
			Febuxostat users (n=2,485)	Allopurinol users (n=598)	Both users (n=159)	
*Demographic and physical profile*						
Age (years)	64.7 ± 15.6	64.1 ± 15.7	67.9 ± 14.6	67.6 ± 14.8	66.3 ± 15.0	*< 0.001*
< 50 years	3,439 (17.7)	3,037 (18.8)	314 (12.6)	66 (11.0)	22 (13.8)	*< 0.001*
50 - 64 years	5,601 (28.9)	4,765 (29.5)	619 (24.9)	170 (28.4)	47 (29.6)	
≥ 65 years	10,349 (53.4)	8,345 (51.7)	1,552 (62.5)	362 (60.5)	90 (56.6)	
Sex (%)						*< 0.001*
Female	8,803 (45.4)	7,850 (48.6)	767 (30.9)	151 (25.3)	35 (22.0)	
Male	10,586 (54.6)	8,297 (51.4)	1,718 (69.1)	447 (74.8)	124 (78.0)	
BMI (Kg/m^2^)	25.0 ± 4.6	24.9 ± 4.5	25.8 ± 4.6	25.7 ± 4.3	25.5 ± 4.2	*< 0.001*
*Comorbidities*						
Hyperuricemia (%)	8,174 (42.2)	6,854 (42.5)	863 (34.7)	374 (62.5)	83 (52.2)	*< 0.001*
Hypertension (%)	15,794 (81.5)	12,760 (79.0)	2,336 (94.0)	550 (92.0)	148 (93.1)	*< 0.001*
Diabetes mellitus (%)	8,314 (42.9)	6,550 (40.6)	1,388 (55.9)	301 (50.3)	75 (47.2)	*< 0.001*
Hyperlipidemia (%)	9,778 (50.4)	7,785 (48.2)	1,564 (62.9)	344 (57.5)	85 (53.5)	*< 0.001*
Chronic kidney disease (%)						*< 0.001*
Stage 1 (%)	3,779 (19.5)	3,719 (23.0)	24 (1.0)	33 (5.5)	3 (1.9)	
Stage 2 (%)	4,895 (25.3)	4,617 (28.6)	173 (7.0)	87 (14.6)	18 (11.3)	
Stage 3a (%)	2,883 (14.9)	2,445 (15.1)	317 (12.8)	95 (15.9)	26 (16.4)	
Stage 3b (%)	2,848 (14.7)	2,082 (12.9)	591 (23.8)	135 (22.6)	40 (25.2)	
Stage 4 (%)	2,231 (11.5)	1,388 (8.6)	670 (27.0)	125 (20.9)	48 (30.2)	
Stage 5 (%)	2,753 (14.2)	1,896 (11.7)	710 (28.6)	123 (20.6)	24 (15.1)	
Any cardiovascular disease (%)	6,411 (33.1)	4,970 (30.8)	1,098 (44.2)	272 (45.5)	71 (44.7)	*< 0.001*
Kidney stones (%)	2,482 (12.8)	2,059 (12.8)	319 (12.8)	83 (13.9)	21 (13.2)	*0.877*
*Frail assessment results*						
FRAIL scale	0.45 ± 0.62	0.42 ± 0.61	0.62 ± 0.68	0.50 ± 0.60	0.46 ± 0.55	*< 0.001*
*Medication usage*						
Anti-hypertensives (%)	12,666 (65.3)	9,960 (61.7)	2,079 (83.7)	491 (82.1)	136 (85.5)	*< 0.001*
ACEi	93 (0.5)	76 (0.5)	12 (0.5)	5 (0.8)	0 (0)	*0.496*
ARB	3,264 (16.8)	2,601 (16.1)	496 (20.0)	133 (22.2)	34 (21.4)	*< 0.001*
CCB	7,826 (40.4)	6,044 (37.4)	1,370 (55.1)	322 (53.9)	90 (56.6)	*< 0.001*
Diuretics	6,028 (31.1)	4,575 (28.3)	1,119 (45.0)	264 (44.2)	70 (44.0)	*< 0.001*
β-blockers	5,511 (28.4)	4,167 (25.8)	1,021 (41.1)	247 (41.3)	76 (47.8)	*< 0.001*
α-blockers	3,075 (15.9)	2,141 (13.3)	737 (29.7)	145 (24.3)	52 (32.7)	*< 0.001*
Anti-diabetics (%)	6,174 (31.8)	4,741 (29.4)	1,143 (46.0)	232 (38.8)	58 (36.5)	*< 0.001*
Anti-lipemics (%)	5,871 (30.3)	4,508 (27.9)	1,064 (42.8)	237 (39.6)	62 (39.0)	*< 0.001*
Anti-platelet agents (%)	5,175 (26.7)	4,019 (24.9)	869 (35.0)	235 (39.3)	52 (32.7)	*< 0.001*
Anti-coagulants (%)	879 (4.5)	667 (4.1)	171 (6.9)	34 (5.7)	7 (4.4)	*< 0.001*
NSAIDs (%)	1,546 (8.0)	1,335 (8.3)	152 (6.1)	46 (7.7)	13 (8.2)	*0.003*
Laboratory data^*^						
Uric acid (mg/dL)	6.40 ± 2.25	6.41 ± 2.21	6.06 ± 2.45	7.42 ± 1.85	6.84 ± 2.46	*< 0.001*
eGFR (mL/min/1.73m^2^)	57.83 ± 37.57	63.06 ± 37.88	29.70 ± 20.14	39.30 ± 27.07	35.98 ± 21.04	*< 0.001*

ACEi, angiotensin-converting enzyme inhibitor; ARB, angiotensin receptor blocker; BMI, body mass index; CCB, calcium channel blocker; eGFR, estimated glomerular filtration rate; NSAID, non-steroidal anti-inflammatory drug * By ANOVA tests

**Table 2:** Incidence and probability of frailty development among participants

Variables	Events	P	PYs	Cumulative incidence	Incidence density*	Crude		Model 1^#^	
						HR	95% CI	HR	95% CI
*UA-lowering treatments*									
Non-users	698	16,147	54,754.5	4.32%	12.75	1.000	-	1.000	-
Febuxostat users	150	2,485	6,318.4	6.04%	23.74	1.992	1.668-2.381^†††^	1.221	1.010-1.477^†^
Allopurinol users	32	598	2,490.9	5.35%	12.85	0.974	0.684-1.389	0.652	0.455-0.933^†^
Both users	12	159	624.6	7.55%	19.21	1.479	0.836-2.617	1.290	0.726-2.293

CI, confidence interval; eGFR, estimated glomerular filtration rate; HR, hazard ratio; P, population; PY, person-year; UA, uric acid ^*^ per 1000 patient-year ^#^ Incorporating demographic and physical data, comorbidities, medications, FRAIL results, and laboratory data (eGFR and UA) ^†^p < 0.05 ^†††^p < 0.001

**Table 3:** Incidence and probability of worsening frailty among participants

Variables	Events	P	PYs	Cumulative incidence	Incidence density*	Crude		Model 1^#^	
						HR	95% CI	HR	95% CI
*UA-lowering treatments*									
Non-users	4,328	16,147	47,337.0	26.80%	91.43	1.000	-	1.000	-
Febuxostat users	652	2,485	5,480.2	26.24%	118.97	1.307	1.203-1.420^†††^	1.051	0.962-1.148
Allopurinol users	213	598	2,066.8	35.62%	103.06	1.126	0.982-1.293	0.919	0.800-1.057
Both users	60	159	524.1	37.74%	114.49	1.250	0.969-1.612	1.019	0.788-1.316

CI, confidence interval; eGFR, estimated glomerular filtration rate; HR, hazard ratio; P, population; PY, person-year; UA, uric acid ^*^ per 1000 patient-year ^#^ Incorporating demographic data, comorbidities, medications, laboratory data (eGFR and UA) ^†††^p < 0.001

**Table 4:** Subgroup analyses results

Variables	Events	P	PYs	Cumulative incidence	Incidence density*	Crude		Model 1^#^	
						HR	95% CI	HR	95% CI
*Incident frailty*									
*Age < 50*									
Non-users	38	3,037	11,169.5	1.25%	3.40	1.000	-	1.000	-
Febuxostat users	11	314	913.1	3.50%	12.05	3.652	1.856-7.188^†††^	1.585	0.768-3.270
Allopurinol users	1	66	315.5	1.52%	3.17	0.909	0.125-6.628	0.933	0.126-6.928
Both users	0	22	99.9	0%	0	0	-	0	-
*Age 50-64*									
Non-users	126	4,765	17,566.0	2.64%	7.17	1.000	-	1.000	-
Febuxostat users	19	619	1,750.7	3.07%	10.85	1.722	1.059-2.801^†^	1.027	0.617-1.709
Allopurinol users	7	170	775.2	4.12%	9.03	1.199	0.599-2.568	0.701	0.322-1.523
Both users	2	47	209.3	4.26%	9.55	1.265	0.312-5.121	1.471	0.356-6.068
*Age ≥ 65*									
Non-users	534	8,345	26,019.0	6.40%	20.52	1.000	-	1.000	-
Febuxostat users	120	1,552	3,654.6	7.73%	32.84	1.716	1.405-2.095^†††^	1.276	1.029-1.583^†^
Allopurinol users	24	362	1,400.2	6.63%	17.14	0.801	0.532-1.206	0.639	0.422-0.968^†^
Both users	10	90	315.3	11.11%	31.71	1.530	0.818-2.861	1.398	0.744-2.629
*Male*									
Non-users	326	8,297	27,694.0	3.93%	11.77	1.000	-	1.000	-
Febuxostat users	82	1,718	4,425.7	4.77%	18.53	1.721	1.348-2.199^†††^	1.188	0.918-1.538
Allopurinol users	20	447	1,869.5	4.47%	10.70	0.862	0.549-1.355	0.630	0.399-0.996^†^
Both users	9	124	493.7	7.26%	18.23	1.485	0.766-2.882	1.563	0.802-3.048
*Female*									
Non-users	372	7,850	27,060.5	4.74%	13.75	1.000	-	1.000	-
Febuxostat users	68	767	1,892.7	8.87%	35.93	2.757	2.125-3.578^†††^	1.222	0.923-1.616
Allopurinol users	12	151	621.4	7.95%	19.31	1.384	0.779-2.459	0.698	0.390-1.249
Both users	3	35	130.9	8.57%	22.92	1.657	0.532-5.162	0.838	0.267-2.631
*eGFR < 45*									
Non-users	372	5,366	16,296.4	6.93%	22.83	1.000	-	1.000	-
Febuxostat users	128	1,971	4,896.8	6.49%	26.14	1.232	1.006-1.508^†^	1.175	0.950-1.455
Allopurinol users	23	383	1,570.2	6.01%	14.65	0.601	0.394-0.917^†^	0.552	0.361-0.844^††^
Both users	10	112	430.6	8.93%	23.22	0.962	0.513-1.804	1.210	0.642-2.282
*eGFR < 30*									
Non-users	225	3,284	9,618.1	7.76%	26.51	1.000	-	1.000	-
Febuxostat users	96	1,380	3,304.9	6.96%	29.05	1.170	0.923-1.483	1.114	0.864-1.435
Allopurinol users	8	248	982.8	3.23%	8.14	0.292	0.145-0.592^††^	0.263	0.129-0.534^†††^
Both users	8	72	273.2	11.11%	29.28	1.075	0.531-2.175	1.364	0.669-2.781

CI, confidence interval; eGFR, estimated glomerular filtration rate; HR, hazard ratio; P, population; PY, person-year; UA, uric acid ^*^ per 1000 patient-year ^#^ Incorporating demographic data, comorbidities, medications, laboratory data (eGFR and UA) ^†^p < 0.05 ^††^p < 0.01 ^†††^p < 0.001

## Data Availability

The raw data for conducting this analysis are unavailable according to the regulatory policy of National Taiwan University Hospital Integrated Medical Database.

## Abbreviations

ANOVA: analysis of variance
BMI: body mass index
CARE: Cardiovascular Safety of Febuxostat and Allopurinol in Patients with Gout and Cardiovascular Morbidities
CI: confidence interval
CKD: chronic kidney disease
CKD-EPI: chronic kidney disease-epidemiology collaboration
eGFR: estimated glomerular filtration rate
FRAIL: fatigue, resistance, ambulation, illness, and loss of weight
HR: hazard ratio
ICD: international classification of disease
IPTW: inverse probability of treatment weighting
MDRD: Modification of Diet in Renal Disease
NSAID: non-steroidal anti-inflammatory drug
NTUH-iMD: National Taiwan University Hospital integrated Medical Database
UA: uric acid
