# Supplementary material for: Differential associations of febuxostat and allopurinol with incident and worsening frailty risk in nephrology patients
Source: Int J Med Sci. 2026 Aug 4;23(9):2963–73. doi: 10.7150/ijms.128982 (PMC13539922; doi:10.7150/ijms.128982)
Supplement: Supplementary file 1 [file ijmsv23p2963s1.pdf]

Supplementary Table S1. Probability of incident and worsening frailty using IPTW

Variables	HR	95% CI	*P value*
Incident frailty			
Non-users	1.000		
Febuxostat users	1.604	1.462 – 1.760	*<0.001*
Non-users	1.000		
Allopurinol users	0.963	0.684 – 1.357	*0.831*
Worsening frailty			
Non-users	1.000		
Febuxostat users	1.045	1.003 – 1.089	*0.036*
Non-users	1.000		
Allopurinol users	1.127	0.988 – 1.287	*0.076*

CI, confidence interval; HR, hazard ratio; IPTW, inverse probability of treatment weighting

Supplementary Table S2. Negative outcome (urinary tract infection) incidence and probability analysis

Variables	Events	P	PYs	Cumulative incidence	Incidence density*	Crude		Model#	
						HR	95% CI	HR	95% CI
UA-lowering treatments									
Non-users	2,292	16,147	50,839.0	14.19%	45.08	1.000	-	1.000	-
Febuxostat users	313	2,485	5,984.8	12.60%	52.30	1.069	0.950-1.203	1.004	0.884-1.139
Allopurinol users	109	598	2,275.9	18.23%	47.89	1.118	0.923-1.355	1.122	0.923-1.364
Both users	26	159	576.0	16.35%	45.13	1.050	0.713-1.545	1.122	0.760-1.654

CI, confidence interval; eGFR, estimated glomerular filtration rate; HR, hazard ratio; P, population; PY, person-year; UA, uric acid

* per 1000 patient-year

Incorporating demographic, comorbidities, medications, FRAIL results, and laboratory data (eGFR and UA)

Supplementary Table S3. Incidence and probability of frailty development and worsening frailty according to the duration of UA-lowering treatment used

Variables	Events	P	PYs	Cumulative incidence	Incidence density*	Crude		Model[#]	
						HR	95% CI	HR	95% CI
Frailty development									
Febuxostat									
Non-users	698	16,147	54,754.5	4.32%	12.75	1.000	-	1.000	-
≤ median duration[a]	81	1,333	3,528.8	6.08%	22.95	1.895	1.504-2.387[†††]	1.152	0.908-1.463
> median duration[a]	81	1,311	3,414.1	6.18%	23.72	1.965	1.559-2.475[†††]	1.287	1.008-1.643[†]
						P for trend <0.001		***P for trend 0.0287***	
Allopurinol									
Non-users	698	16,147	54,754.5	4.32%	12.75	1.000	-	1.000	-
≤ median duration[b]	25	416	1,694.6	6.01%	14.75	1.128	0.757-1.682	0.852	0.570-1.275
> median duration[b]	19	341	1,420.9	5.57%	13.37	1.011	0.641-1.596	0.626	0.395-0.992[†]
						P for trend 0.7538		***P for trend 0.0351***	
Worsening frailty									
Febuxostat									
Non-users	4,328	16,147	47,337.0	26.80%	91.43	1.000	-	1.000	-
≤ median duration[a]	344	1,333	3,049.3	25.81%	112.81	1.237	1.109-1.381[†††]	0.995	0.889-1.115
> median duration[a]	368	1,311	2,955.0	28.07%	124.54	1.367	1.229-1.521[†††]	1.102	0.984-1.234
						P for trend <0.001		*P for trend 0.1451*	
Allupurinol									

Non-users	4,328	16,147	47,337.0	26.80%	91.43	1.000	-	1.000	-
≤ median duration[b]	135	416	1,451.8	32.45%	92.99	1.014	0.854-1.204	0.843	0.709-1.003
> median duration[b]	138	341	1,139.0	40.47%	121.15	1.325	1.118-1.570[††]	1.016	0.855-1.206
						P for trend 0.0037		*P for trend 0.4874*	

CI, confidence interval; eGFR, estimated glomerular filtration rate; HR, hazard ratio; P, population; PY, person-year; UA, uric acid

[*] per 1000 patient-year

[#] Incorporating demographic, comorbidities, medications, FRAIL results, and laboratory data (eGFR and UA)

[a]161 days; [b]140 days

[†]$p < 0.05$; [††]$p < 0.01$; [†††]$p < 0.001$

Supplementary Table S4. Incidence and probability of frailty development and worsening frailty

Variables	Incidence density*	Crude		Model 2[@]		Model 3[%]		Model 4[&]	
		HR	95% CI	HR	95% CI	HR	95% CI	HR	95% CI
Frailty development									
Non-users	12.75	1.000	-	1.000	-	1.000	-	1.000	-
Febuxostat users	23.74	1.992	1.668-2.381[†††]	1.183	0.977-1.433[a]	1.187	0.981-1.435[b]	1.086	0.894-1.320
Allopurinol users	12.85	0.974	0.684-1.389	0.642	0.448-0.919[†]	0.638	0.445-0.913[†]	0.713	0.493-0.999[†]
Both users	19.21	1.479	0.836-2.617	1.259	0.708-2.239	1.262	0.710-2.243	1.310	0.735-2.335
Worsening frailty									
Non-users	91.43	1.000	-	1.000	-	1.000	-	1.000	-
Febuxostat users	118.97	1.307	1.203-1.420[†††]	1.041	0.953-1.138	1.017	0.930-1.111	0.972	0.888-1.064
Allopurinol users	103.06	1.126	0.982-1.293	0.917	0.797-1.054	0.902	0.784-1.037	0.933	0.814-1.069
Both users	114.49	1.250	0.969-1.612	1.019	0.788-1.317	0.997	0.772-1.288	1.047	0.820-1.337

ACEi, angiotensin-converting enzyme inhibitor; ARB, angiotensin receptor blocker; CCB, calcium channel blocker; CI, confidence interval; CKD-EPI, Chronic Kidney Disease-Epidemiology Collaboration; eGFR, estimated glomerular filtration rate; HR, hazard ratio; UA, uric acid

[*] per 1000 patient-year

[@] Incorporating Model 1 variables, with blood pressure-lowering drugs divided according to types

[%] Incorporating Model 1 variables, but eGFR calculated using the CKD-EPI formula

[&] Incorporating Model 1 variables, using death as competing risk event, in combination with Models 2 and 3 variables

[†]*$p < 0.05$*; [†††]*$p < 0.001$*

[a] $p = 0.085$; [b] $p = 0.077$; [c] $p = 0.062$
